# The Appropriateness of Invasive Ventilation in COVID-19 Positive Cancer Patients: Proposal of a New Prognostic Score

**DOI:** 10.3390/v13030508

**Published:** 2021-03-19

**Authors:** Michele Ghidini, Alice Indini, Erika Rijavec, Claudia Bareggi, Monica Cattaneo, Gianluca Tomasello, Barbara Galassi, Donatella Gambini, Francesco Grossi

**Affiliations:** Medical Oncology Unit, Fondazione IRCCS Ca’ Granda Ospedale Maggiore Policlinico, 20122 Milan, Italy; michele.ghidini@policlinico.mi.it (M.G.); alice.indini@policlinico.mi.it (A.I.); erika.rijavec@policlinico.mi.it (E.R.); claudia.bareggi@policlinico.mi.it (C.B.); aprile83@gmail.com (M.C.); gianluca.tomasello@policlinico.mi.it (G.T.); barbara.galassi@policlinico.mi.it (B.G.); donatella.gambini@policlinico.mi.it (D.G.)

**Keywords:** COVID-19, invasive ventilation, cancer, ICU, ONCOVID-ICU, Milano Policlinico, SOFA score, ARDS

## Abstract

Over the last months, as oncology specialists, we have frequently been contacted for estimating prognosis for cancer patients affected by COVID-19 infection. Until now, there have been no clear markers to guide decision making regarding the appropriateness of invasive ventilation in cancer patients affected by COVID-19 infection. We developed a practical tool encompassing a prognostic score, “The Milano Policlinico ONCOVID-ICU score.” The score is composed of three groups of variables: patient’s characteristics such as sex, age, BMI, and comorbidities; oncological variables (treatment intent, life expectancy, on or off-treatment status); and clinical parameters in association with laboratory values (the Sequential Organ Failure Assessment (SOFA) score and D-dimer). The SOFA score includes six different clinical parameters and during the first few days of ICU admissions has an important prognostic role. The oncological history should never represent, per se, a contraindication to intensive care and must be considered together with other variables, such as laboratory values, clinical parameters, and patient characteristics, in order to make the hardest but best possible choice. To our knowledge, “The Milano Policlinico ONCOVID-ICU score” is the first prognostic score proposed in this setting of patients and requires further validation. This tool may be useful to assess the prognosis of cancer patients in critical conditions.

## 1. Introduction

Over the last two months, as oncology specialists, we have frequently been contacted to estimate prognosis and proactively make treatment escalation plans for cancer patients affected by COVID-19 infection. In the case of acutely deteriorating patients, such decisions often have to be made within a short space of time and can be challenging. Our Hospital Maggiore Policlinico of Milan is located in the center of Milan and has a large emergency room, thus being able to accommodate patients from many different hospitals that do not have access to such services. Since the pandemic began, our hospital has been totally restructured in order to cope with the increased caseload. So far, patients with COVID-19 and its complications have occupied more than 500 beds (half of the total bed capacity). 

One Wednesday morning, we were contacted by a colleague who was caring for patients with COVID-19 in a different hospital in Milan. He wanted to discuss the ceiling of treatment for a mutual patient who was admitted with acute respiratory failure secondary to COVID-19-induced pneumonia and had increasing oxygen requirements. Is invasive mechanical ventilation appropriate in patients with active cancer? Our colleague instinctively proposed a palliative strategy without the use of C-PAP or invasive ventilation. Why? Our 65-year-old patient had a history of recurrent pancreatic cancer treated with single-agent capecitabine for six months after eight cycles of second-line chemotherapy with capecitabine and oxaliplatin and disease stability. Her progression-free interval from the beginning of second-line therapy is approaching one year, overcoming our expectations and average median survival. She was last reviewed in our outpatient clinic on 13 March when she was well with an ECOG performance status (PS) of zero and no treatment-related toxicities to report. 

We only had a short moment to gather our thoughts and remind ourselves of the patient’s clinical case. Some minutes later, we were expected to make the ultimate decision for our patient: was further ventilatory support appropriate or not? Was our colleague right to suggest palliative care only? We took into consideration her age, good PS before COVID-19 infection, lack of significant comorbidities, the unusually long progression-free interval, suggesting favorable disease biology, and the good tolerability of her recent chemotherapy. We recommended full escalation of treatment from C-PAP to invasive ventilation if deemed necessary. Were we wrong? We were not aware of any tools to support us with this tough decision. 

## 2. Invasive Ventilation in Cancer Patients

It has been reported that the mortality rate of cancer patients admitted to European intensive care units (ICUs) is similar to that of those without cancer (20% vs. 18%, respectively). However, medical patients with advanced cancer have double the hospital mortality rate of surgical patients with cancer (41 vs. 21%, *p* < 0.001) [1]. Prospective observational data showed that even high-risk cancer patients may benefit from early admission to ICUs, especially if treated before the onset of organ dysfunction. In these patients, a “full-code management” without limitations of ICU resources should be done for the first days (ideally ≥5 days) because prognosis cannot be estimated until this monitoring period has passed [2,3]. A recent systematic review attempted to establish a consensus on indications for intubation in cancer patients. Over the years, there has been an improvement in the outcome of cancer patients admitted to the ICU, with an average survival of 32.4% and a long-term survival of 10.2% [4]. Among the risk factors for short-term mortality, such as age, severity, and the number of failing organs, the presence of acute respiratory failure, PS, comorbidities, and stage of the disease play a fundamental role [3,4]. Moreover, the Sequential Organ Failure Assessment (SOFA) score was reported as one of the major predictors of outcome for cancer patients admitted to ICU [4]. The SOFA score includes six different clinical parameters: the respiratory PaO2/FIO2 (P/F) ratio, presence of ventilatory assistance, blood pressure, platelet count, the Glasgow Coma Score scale, and bilirubin and creatinine levels (Table 1). 

Sequential evaluation of the SOFA score during the first few days of ICU admissions has an important prognostic role. In the case of a stable or increase in the SOFA score in the first hours after admission to ICU, the reported death rates are 37% for a score between 2 and 7, 60% with an initial score of 8–11, and 91% in case of a score > 11. On the contrary, for initial scores < 11, a decreasing value is associated with a mortality rate of 6% [5]. 

Among predictors of poor outcome, the etiology of respiratory failure must be taken into consideration as well. While pulmonary edema in cancer patients has a reasonably good prognosis, infection is a predictor of poor outcome [4]. COVID-19 infection and subsequent pneumonitis can cause a condition of hypoxiemic respiratory failure and acute respiratory distress syndrome (ARDS), requiring oxygen and ventilation therapies, admittance to ICU in 15–20% of cases, and, in approximately 3.2% of cases, intubation and invasive ventilation [6]. A prospective Chinese series of COVID-19 positive patients reported a statistically significant increase in terms of severe events requiring ICU admittance, invasive ventilation, or death in cancer patients compared with cancer-free cases (39 vs. 8%, p = 0.0003). Moreover, patients undergoing surgery or chemotherapy for cancer in the previous months had a higher risk of clinically severe events when compared to patients who did not have these treatments (75 vs. 43%) [7]. Risk factors for mortality in adult inpatients with COVID-19 have been described in a Chinese retrospective multicenter cohort study. The median age of the population was 56 years. Multivariable regression identified older age (odds ratio (OR) = 1.10, p = 0.0043), a higher SOFA score (OR 5.65, p < 0.0001) and a D-dimer greater than 1 μg/mL (OR 18.42, p = 0.0033) on admission as significant prognostic factors for survival [8].

Identification of prognostic factors for critically ill cancer patients following infection with COVID-19 is challenging but essential in order to define appropriate candidates for ICU admission and intubation. Many variables are present and most of the available series are mainly from the Chinese experience. The total number of cases, number of cases admitted to ICUs, median age of diagnosis, and mortality rates are far different from what was registered in Western European countries. Globally, the median age of patients diagnosed with COVID-19 infection in Europe is higher than in China. Moreover, at the time of writing, Spain, Italy, the UK, and France have registered a mortality rate of between 10 and 13% compared to approximately 5% registered in China, while the rate of ICU admission remains similar at 2–3% [9]. These differences may be justified at least partially by a higher number of tests performed in China and the difference in the median age of the population. Age is an important factor to take into consideration before admitting a cancer patient into the ICU. However, chronological age alone is a poor indicator of the physiological and functional status of a cancer patient and should not be considered as the main prognostic factor for treatment decisions in oncology [10]. Indeed, many patients over the age of 70 have an excellent PS and are suitable candidates for oncological treatment. 

## 3. Results

Until now, there have been no clear markers to guide decision making regarding the appropriateness of invasive ventilation in cancer patients affected by COVID-19 infection. Therefore, we developed a practical tool that encompasses a prognostic score in order to identify a subgroup of patients likely to have a better outcome and therefore may be potential candidates for invasive ventilation. The Milano Policlinico ONCOVID-ICU score includes three different groups of variables. In the first group, we include sex, age, body mass index (BMI), and comorbidities. In a previous series, male sex was identified as an independent risk factor associated with worse prognosis and lack of clinical improvement in COVID-19 patients admitted to hospital (OR = 0.486, p = 0.001) [11]. Moreover, we included BMI and comorbidities as they can be limiting factors for oncological treatment and were reported as risk factors for short-term mortality in critically ill cancer patients requiring invasive ventilation [3,4]. Old age was reported as one of the poor prognostic factors for survival in COVID-19 inpatients [8]. The second group includes oncological variables, such as the treatment intent (adjuvant or metastatic), life expectancy in months, and availability of further treatment lines. Furthermore, we included the SOFA score and the D-dimer values, previously reported as risk factors for mortality in the presence of COVID-19 infection [8]. We identified three different groups (low, intermediate, and high risk). We recommend that patients with a low risk score should be offered invasive procedures if necessary, while high-risk patients are best managed with the best supportive care. Patients in the intermediate-risk group deserve a case-by-case discussion to derive a decision (Table 2). 

## 4. Discussion

The prognosis of COVID-19 patients has improved so far because of the reinforced clinical experience and the advent of new treatment options, such as antivirals, antibiotics, convalescent plasma, and monoclonal antibodies [12]. Intensive care management is particularly complex, with the management of acute respiratory failure and hemodynamic support being key [12]. This division in the category of risks that we adopted is arbitrary, and the score needs further validation. We aim to validate our score by retrospectively assessing the clinical history of all cancer patients admitted to Milano Hospital Maggiore Policlinico’s ICU. 

Cancer patients are facing higher risks of COVID-19 infection compared to the general population, with increased susceptibility to long-term infections and higher mortality [13,14]. Uncertainty regarding the safety of treatments (e.g., immunotherapy) in times of infection is a major topic of discussion. Furthermore, a considerable proportion of oncology patients may experience clinical deterioration due to the worsening course of the infection. These cases require a comprehensive evaluation before considering ICU admission and intubation. In addition, ethical issues on withholding care for patients have always to be considered. The Milano Policlinico ONCOVID-ICU score may help physicians to make a decision about critical clinical cases. However, patients and their relatives keep the right to be informed and the authority for final decision making. The oncological history should never represent, per se, a contraindication to intensive care and must be considered together with other variables, such as laboratory values, clinical parameters, and patient characteristics, in order to make the hardest but best possible choice.

## Figures and Tables

**Table 1:** The Sequential Organ Failure Assessment (SOFA) score.

Variables	SOFA Score				
	0	1	2	3	4
**Respiratory** PaO_2_/FIO_2_ (P/F) mmHg	>400	≤400	≤300	≤200 *	≤100 *
**Coagulation** Platelets x 10^9^/μg	>150	≤150	≤100	≤50	≤20
**Liver** Bilirubin mg/dL	<1.2	1.2–1.9	2.0–5.9	6.0–11.9	>12
**Cardiovascular** Hypotension	No hypotension	Mean arterial pressure < 70 mmHg	Dopamine ≤ 5 or dobutamine (any dose)	Dopamine > 5, epinephrine ≤ 0.1, or norepinephrine ≤ 0.1	Dopamine > 15, epinephrine > 0.1 or norepinephrine > 0.1
**Central nervous system** Glasgow Coma Score Scale	15	13–14	10–12	6–9	<6
**Renal** Creatinine mg/dL	<1.2	1–2–1.9	2.0–3.4	3.5–4.9	>5

* with ventilatory support.

**Table 2:** The Milano Policlinico ONCOVID-ICU score.

**Variables**	**Score**	**Categories of risk for patients ** **Score < 4: Low Risk ** ICU admission and invasive ventilation. **Score 4–6: Intermediate Risk ** Case-by-case evaluation for appropriateness of ICU admission and invasive ventilation. **Score ≥ 7: High Risk ** Palliative treatment.
**Patient characteristics ** Sex Age BMI Comorbidities	F = 0 M = 1 < 70 = 0 ≥ 70 = 1 < 30 = 0 ≥ 30 = 1 NO = 0 YES = 1 YES > 1 = 2	
**Oncological variables ** Treatment intent Life expectancy Patient on treatment	Curative = 0 Palliative = 1 > 6 mo = 0 < 6 mo = 1 NO = 0 YES = 1	
**Clinical parameters + laboratory values ** SOFA SCORE D-dimer	2–7 = 0 ≥ 8 = 1 < 1 μg/mL= 0 >1 μg/mL = 1	

## References

[1] Taccone F.S., Artigas A.A., Sprung C.L., Moreno R., Sakr Y., Vincent J.L. (2009). Characteristics and outcomes of cancer patients in European ICUs. Crit. Care.

[2] Lecuyer L., Chevret S., Thiery G., Darmon M., Schlemmer B., Azoulay É. (2007). The ICU Trial: A new admission policy for cancer patients requiring mechanical ventilation. Crit. Care Med..

[3] Schellongowski P., Sperr W.R., Wohlfarth P., Knoebl P., Rabitsch W., Watzke H.H., Staudinger T. (2016). Critically ill patients with cancer: Chances and limitations of intensive care medicine—A narrative review. ESMO Open.

[4] Huaringa A.J., Francis W.H. (2019). Outcome of invasive mechanical ventilation in cancer patients: Intubate or not to intubate a patient with cancer. J. Crit. Care.

[5] Ferreira F.L. (2001). Serial Evaluation of the SOFA Score to Predict Outcome in Critically Ill Patients. JAMA.

[6] Meng L., Qiu H., Wan L., Ai Y., Xue Z., Guo Q., Deshpande R., Zhang L., Meng J., Tong C. (2020). Intubation and Ventilation amid the COVID-19 Outbreak. Anesthesiology.

[7] Liang W., Guan W., Chen R., Wang W., Li J., Xu K., Li C., Ai Q., Lu W., Liang H. (2020). Cancer patients in SARS-CoV-2 infection: A nationwide analysis in China. Lancet Oncol..

[8] Zhou F., Yu T., Du R., Fan G., Liu Y., Liu Z., Xiang J., Wang Y., Song B., Gu X. (2020). Clinical course and risk factors for mortality of adult inpatients with COVID-19 in Wuhan, China: A retrospective cohort study. Lancet.

[9] Statista From COVID-19/Coronavirus, Facts and Figures. https://www.statista.com/page/covid-19-coronavirus.

[10] Soto-Perez-de-Celis E., Li D., Yuan Y., Lau Y.M., Hurria A. (2018). Functional versus chronological age: Geriatric assessments to guide decision making in older patients with cancer. Lancet Oncol..

[11] Zhang J., Wang X., Jia X., Li J., Hu K., Chen G., Wei J., Gong Z., Zhou C., Yu H. (2020). Risk factors for disease severity, unimprovement, and mortality of COVID-19 patients in Wuhan, China. Clin. Microbiol. Infect..

[12] Phua J., Weng L., Ling L., Egi M., Lim C.M., Divatia J.V., Shrestha B.R., Arabi Y.M., Ng J., Gomersall C.D. (2020). Intensive care management of coronavirus disease 2019 (COVID-19): Challenges and recommendations. Lancet Respir. Med..

[13] (2020). COVID-19 More Frequent, Severe in Cancer Patients. Cancer Discov..

[14] (2021). COVID-19 Prolonged in Patients with Cancer. Cancer Discov..

